# Sensorimotor synchronization to rhythm in an experienced sea lion rivals that of humans

**DOI:** 10.1038/s41598-025-95279-1

**Published:** 2025-05-01

**Authors:** Peter F. Cook, Carson Hood, Andrew Rouse, Colleen Reichmuth

**Affiliations:** 1 New College of Florida, Sarasota, FL 34243 USA; 2 Institute of Marine Sciences, Long Marine Laboratory, University of California Santa Cruz, Santa Cruz, CA 95060 USA

**Keywords:** Psychology, Sensorimotor processing

## Abstract

Is human beat keeping unique among vertebrates? The only non-human data showing consistent and lagless beat matching to novel stimuli, including music, come from *Ronan*, a trained sea lion [Cook et al., *J. Comp. Psychol.*, 127(4):412–427]. *Ronan’s* convincing demonstration of adaptive auditory-motoric entrainment at age 3 years showed stronger tempo-phase relationships and higher variability than reported in similar studies of human subjects. This apparent performance mismatch has been used to suggest the mechanisms underlying her beat keeping ability are not isomorphic with those of humans. However, in the twelve years since our original report, *Ronan* has continued intermittent beat-keeping practice. Comparative arguments should consider her improved performance with increased experience and evaluate her ability against human subjects performing similar rhythmic tasks. Here, we report *Ronan’s* contemporary ability to synchronize head movements with novel metronomic sounds presented at novel tempos. We also provide data for ten humans moving in time to the same stimuli using a comfortable arm motion with similar amplitude. This sea lion’s sensorimotor synchronization was precise, consistent, and indistinguishable from or superior to that of typical adults. These findings challenge claims of unique neurobiological adaptations for beat keeping in humans.

## Introduction

Despite decades of research with a wide range of species, there is no broad scientific consensus on whether or not human beat keeping – that is perceiving and moving to an isochronous pulse in complex stimuli such as music^1^-- is underpinned by unique biological mechanisms^2^. The most thorough comparative datasets on sensorimotor synchronization actually come from invertebrate animals, with some insects such as fireflies and crickets demonstrating rate-sensitive synchrony with species-appropriate signals^3–5^. The precision and tempo range of these insects rival the performance of humans synchronizing to rhythmic cues. However, unlike humans, who are florid synchronizers with a general ability to hear and move in time to a steady beat in a wide range of stimuli including music, invertebrate synchronizers show functional fixity, only entraining to a narrow range of specific cues. In addition, under the right circumstances, their entrainment behavior is obligate^6^. Although the dynamics of their behavior may match those of humans in laboratory button pressing tasks, they do not show the flexible beat-keeping that humans are capable of. In the laboratory, some birds and mammals show greater behavioral plasticity, demonstrating the ability to move in time (or not) to arbitrary (i.e., ecologically irrelevant and unfamiliar) rhythmic cues, including those found in music^7^. However, most higher vertebrates do not show much evidence of spontaneous auditory-motor synchronization to biologically non-relevant stimuli such as music. Recent integrative reviews^8^ emphasize apparent mismatches between the character of bird and mammal beat keeping relative to the performances of humans to suggest that human beat keeping is subserved by neurobiological mechanisms unique among higher vertebrates.

Most laboratory evidence of beat keeping in non-human vertebrates comes from psittacines, which tend not to show the same degree of consistency and precision as do humans, and from other primates, which seem to have great difficulty with lagless beat keeping^9,10^. The notable exception is *Ronan* the sea lion, who was operantly conditioned to entrain a continuous head bob movement with metronomic sounds, and then demonstrated transfer of this behavior to novel acoustic tempos and wholly novel stimuli, including music^11,12^. *Ronan’s* unprecedented beat keeping behavior was both consistent and relatively precise; there are no empirical data from a non-human mammal or bird that come close in terms of precision and consistency. However, she showed a fairly strong phase-tempo relationship such that her rhythmic motion lagged slightly behind faster tempos and was moderately far ahead of slower tempos. Although similar patterns have been observed in humans^13^, in many laboratory studies, adult humans tend to show a slight negative onset asynchrony^14^–that is, the inflection point of their movements occurs just before the beat, across a range of tempos. In addition, *Ronan’s* initially reported data, obtained when she was 3 years old (prepubertal), show slightly higher variability than is typically observed in human laboratory studies^9^. Both Patel^15^ and Greenfield and Merker^8^ have accordingly suggested that sea lion *Ronan’s* performance, while impressive, may rely on different underlying neurobiological mechanisms. Patel^15^ further emphasizes the extensive history of training required by *Ronan* to acquire such adaptable beat keeping performance, with this presented in contrast to the supposedly naturally emerging and fairly effortless beat keeping responses of humans.

The field of comparative rhythm research, a domain of what has been termed ‘biomusicality’^16^, is at a turning point, with active debate as to whether there is value in continuing to pursue non-human vertebrate laboratory studies given the possibility that what animals may do differs fundamentally from human behaviors of interest. Novel arguments from an evolutionary perspective suggest closer parallels between selective pressures on certain insects and on early hominids that may have led to enhanced temporal abilities and a predisposition for social entrainment^8^. However, given sparse empirical data, the question of parity between human beat keeping and that of non-human mammals and birds remains open. *Ronan* the most consistent and precise mammalian beat keeper in experimental care, has a privileged place in this debate. Given additional experimental work, would *Ronan’s* capability for beat keeping rival that of typical humans? Detailed comparisons of her initial experimental data^11^ to that gathered from human studies^[e.g. 27^ are worthwhile but far from dispositive for two primary reasons. First, *Ronan* has continued to practice beat keeping and synchronization intermittently over the last twelve years, and her current ability as a mature adult should be assessed and compared to that of humans. Second, comparisons to *Ronan’s* precision and phase-tempo relationships have solely used fine-scale haptic tasks performed by humans, such as button- or key-pressing, which differ substantively in form from *Ronan’s* high-amplitude head movements. Theoretical claims regarding behavioral parallels and dysjunctions^1,6,12^ should be made with matched modality “apples-to-apples” comparisons that consider experience, capacity, and expression of rhythmic behavior.

## Rhythmic apprenticeship

Numerous theoreticians have emphasized the extent of training required for sea lion *Ronan* to learn to beat keep^15^. This factor is typically used to contrast the effortless and formally untrained nature of human beat keeping. This framing overstates *Ronan’s* task-specific training history and understates the important role of learning in human rhythmic behavior.

The amount of training conducted before *Ronan* demonstrated reliable entrainment to metronomic stimuli at multiple tempos was not particularly extensive as far as complex laboratory research training goes. As detailed in our original report^11^, *Ronan* quickly learned to move her head continuously while an auditory stimulus was presented. Approximately 30 training sessions followed with each including presentations of two training tempos (80 and 120 bpm); experimenters reinforced her when her bobbing rate moved closer to the stimulus rate. However, *Ronan* made little to no progress toward synchronization or entrainment during this phase of training, instead just producing a highly variable bob rate. Next, the two tempos were isolated in separate sessions, and the reward criterion was switched to one-bob synchronization, such that she was reinforced anytime her head movement came down right on a beat. The criterion was approximated up, step-by-step, to 20 consecutive beats in sessions with either the 80 or 120 bpm stimulus tempo. This teaching period, when *Ronan* showed rapid improvement in performance, took place in fewer than 30 sessions in less than two months. Over the course of about 25 more sessions, Ronan participated in sessions containing both training tempos intermixed, with the same 20-bob criterion applied to each trial. After this, *Ronan* demonstrated spontaneous tempo-flexible entrainment, reliably and precisely, to novel tempo metronomic stimuli. That is, in no more than 90 training sessions, spread over about six months, *Ronan* learned to adaptively move in time to an isochronous stimulus. The bulk of task learning emerged across only 25 sessions. Many complex cognitive tasks take months (or even years) for animals to learn with much denser and more regular training than *Ronan* received, and often with more directly trained stimulus exemplars^17^. We do not believe it is accurate to frame this training as extensive or even particularly unusual. It is an open question whether *Ronan* acquired the ability to entrain during this time, or whether she already had the innate capability and the training served to instruct her to deploy that ability reliably.

Despite the vague statements that abound in the literature, it is simply not true that human beat keeping emerges without explicit and extended learning^18^. Outside of formal music training (which is actually quite common across human cultures) it is true that humans do not receive specific operant training to beat keep. However, humans do show evidence of extended learning in their rhythmic behavior. Wilson and Cook^19^ dubbed this a “rhythmic apprenticeship,” and the evidence is extensive and largely non-controversial. Human infants, toddlers, and young children display poor auditory-motor entrainment^20^. Although there is evidence that human babies can discern rhythmic from non-rhythmic stimuli^21,22^, reliable, consistent, fairly precise beat keeping, of the kind *Ronan* learned over about two months during her adolescence, does not emerge until fairly late in childhood^18^. Of course, this can be explained in part by lack of motor control in these individuals, who may not be able to precisely express an intent to synchronize. Regardless, during these important developmental periods, children are exposed extensively to music, and not just passively. Musical games and exposure to rhythmic poetry and songs are ubiquitous cross-culturally in early childhood enculturation. Further, the rhythms to which children are exposed and the contexts in which they are presented clearly matter. Older children and adults from cultures with different musical traditions involving distinct rhythmic structures show clear evidence of rhythmic enculturation^23–25^. There is even evidence that negative phase offset patterns seen in adults emerge over time, with greater phase-tempo relationships in young children^26,27^. Even this apparent hallmark of human beat keeping is not obligate in adults: musicians tend to show more precise beat-keeping behavior, without this negative offset. Once again, training (or experience) matters.

Adult human beat keeping feels obligate and, at least with relatively simple and culturally familiar rhythms, effortless. But it does not emerge full-fledged in older children without extensive prior learning. Indeed, when one considers the extent of musical exposure and rhythmic games (informal and formal), that typical humans experience during development, *Ronan’s* history of training is remarkable not for being extensive, but for the exact opposite: for how sparse it is, and how late in development it came. Physical maturation in sea lions happens around age 5 to 6 years^28^ so potential postpartum neural plasticity is likely to be most pronounced in the first year or two of life. *Ronan* did not begin rhythmic training until she was three years old, far later during her relevant developmental trajectory than the point at which people begin their rhythmic apprenticeship (which really begins in utero^29^). This point is important for two reasons: *Ronan’s* relatively limited initial training did not exceed relevant rhythmic exposure in adult humans, and she may have continued to improve since her initial training and testing at age 3 years. Thus, a meaningful comparison between adult humans, who are highly experienced rhythmic experts, and sea lion *Ronan*, now age 15 years, should consider her most recent capabilities.

## Comparable behavioral paradigms

While it bears emphasis, Ronan’s rhythmic apprenticeship has been discussed previously^19^. Far less attention has been given to differences between Ronan’s high-amplitude head bobbing response to acoustic tempos and the fine-scale haptic responses used in most human rhythm studies. Certainly, either response modality provides a window into an individual’s capability for entraining body movements to external stimuli. However, the dynamics of the stimulus-matching response might be quite different depending on movement amplitude and motor system.

Button and key pressing tasks have predominated in human rhythm studies^30^. Understandably so, given the simplicity and ease of integration of these responses into computerized data collection setups. However, humans use multiple motor systems, at both fine and gross scales, to move to rhythmic sounds and we have far fewer data regarding consistency, precision, and phase-tempo relationships with larger body movements. This distinction with respect to response amplitude is important if fair comparisons between humans and other animals are to be made. Sea lions do not have an easily accessible motor system with the fine-grained control of the human hand. Humans, however, have motor systems analogous to the sea lion’s head and neck apparatus, perhaps most obviously, the arm. Indeed, sea lions use their necks much like an arm in grabbing tasks. The human head and neck apparatus also has interesting parallels to that of a sea lion, as humans do often bob their heads to music. The amplitude of that movement is, however, much smaller than the bobbing Ronan has learned to produce (30–40 cm top-to-bottom).

The limited available data on human auditory-motoric entrainment with high-amplitude gross motor systems shows much greater variability than button pressing data^31,32^, with higher phase angles that may extend into the positive range. Intuitively, such movements may be more prone to variability and may be more constrained to a tighter “preferred” or kinematically optimal tempo range^33^. This raises the point that the apparent strength of *Ronan’s* phase-tempo relationship in her initial data may have been due in part to the amplitude and related dynamics of the motor response that was trained. While under-remarked in the comparative literature, humans also show evidence of phase-tempo relationships, even in button-pressing tasks; the angular spread of responses relative to stimulus onset is fairly constrained, but it is worth considering whether gross motor responses might amplify these stimulus-driven relationships.

## Current study

**Fig. 1:**
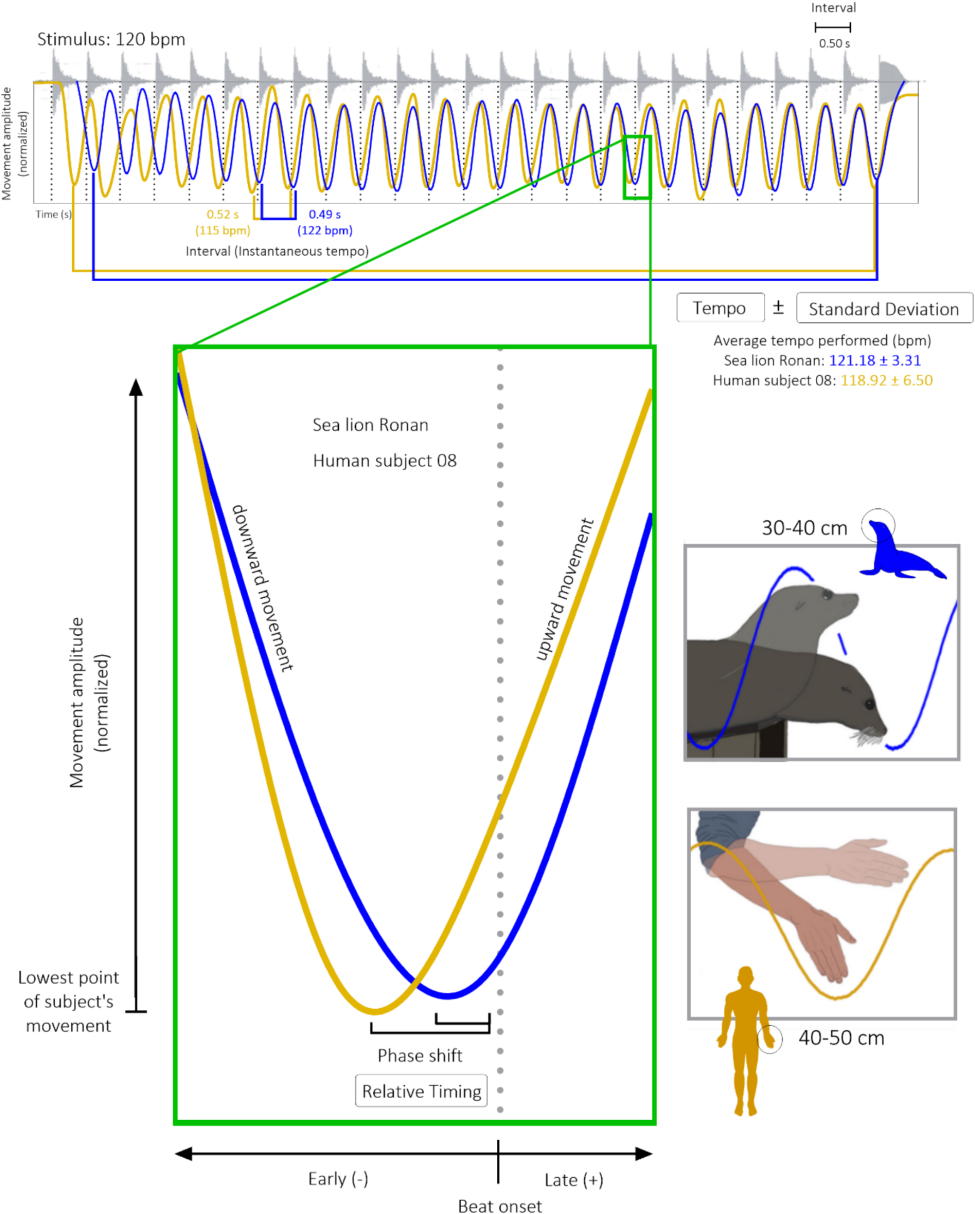
Movement patterns for sea lion *Ronan* and human subject 08 to a 120 bpm metronome. *Ronan* was previously trained through operant conditioning techniques to move her head in tandem with a rhythmic stimulus. For comparison, human subjects were asked to move their preferred arm in a downward, ‘chop’ motion with a similar movement amplitude to *Ronan* to the same rhythmic stimulus. The goal for both subject types was to match the lowest point of their movement with each beat onset. Video tracking software was used to depict movement amplitude over time for both subjects, and significant performance metrics are illustrated.

Here, twelve years after her initial training and testing as reported by Cook et al.^11^, we re-exposed sea lion *Ronan* to the same rhythmic, metronomic stimuli at familiar tempos, 80, 96, 108, and 120 bpm, and compared these results to her original performance. We also assessed *Ronan’s* consistency, precision, and phase-angle relationships with exposure to similar but novel stimuli at previously unexposed tempos, 112 and 128 bpm, evenly spaced around 120 bpm, *Ronan’s* most commonly exposed tempo. We then presented the same stimuli to ten human participants and tasked them with keeping the beat with lower arm movements similar in amplitude and dynamics to *Ronan’s* head bob behavior (see Fig. 1).

We compared *Ronan’s* performance to that of the adults we tested, providing the first apples-to-apples comparative assessment of rhythmic entrainment between humans and non-humans with established beat-keeping ability.

## Results

### Comparison to previous performance

**Fig. 2:**
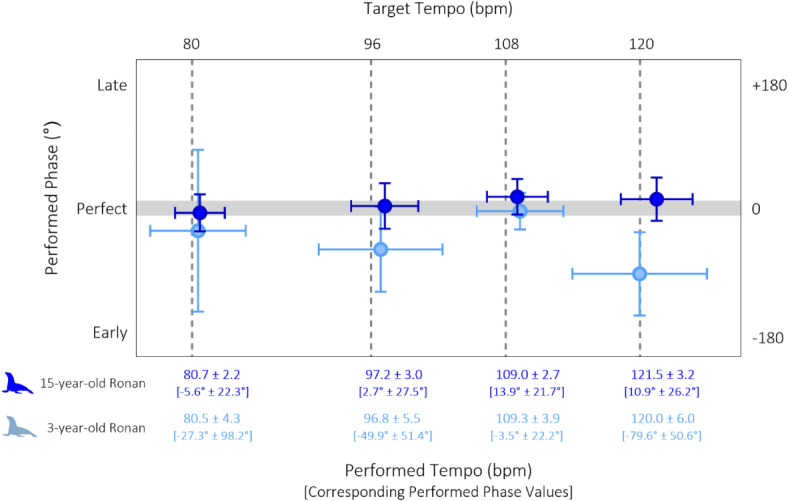
Sea lion *Ronan’s* beat-keeping performance with simple stimuli: comparison of early performance (age 3) versus performance following longitudinal experience with a range of rhythmic stimuli (age 15). The horizontal axes show the exact tempo of the stimulus (top) relative to the sea lion’s performed tempo as mean ± SD (bottom). The vertical axis represents the phase shift in degrees between the nadir of the response and the onset of each beat as mean ± SD, with the top and bottom of the plot anchored at + 180 degrees and − 180 degrees from beat onset. Phase shift values are listed under their corresponding performed tempo. Note that by age 15, *Ronan* consistently shows near perfect tempo and phase in every condition.

Compared to her performance at age 3 years, *Ronan’s* bobbing was more consistent and precise at 15 years old at the measured rhythms of 80, 96, 108, and 120 bpm (see Fig. 2; Table 1, Supplemental Video S1, https://youtu.be/oen67G5w5S0).

**Table 1:**
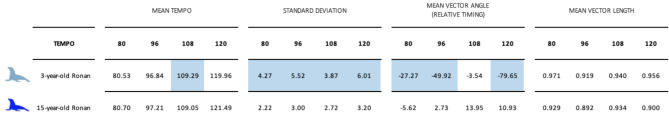
Averages of each metric across 4 stimulus exposures, per subject per stimulus tempo. Each instance where 15-year-old sea Lion *Ronan* performed movements that were closer to perfect synchrony than 3-year-old *Ronan* is highlighted in Blue.

With the exception of 108 bpm, where her mean phase was already close to 0 degrees at initial testing (indicating perfect synchronization), her phase-tempo offset across the measured stimulus rates reduced to near-perfect. Interval standard deviation and phase standard deviation also decreased across all stimulus presentation rates. Ronan’s mean phase angle values were significantly closer to zero from those at initial testing for each stimulus rate except at 108 bpm where she was slightly worse (80 bpm, F = 114, *P* < 0.0001; 96 bpm, F = 67.92, *P* < 0.0001; 108 bpm, F = 5.74, *P* = 0.019; 120 bpm, F = 43.77, *P* < 0.0001). Importantly, *Ronan* produced exactly one bob per beat on all test trials, with no extra head bobs between beats.

### Comparison to human performance

**Fig. 3:**
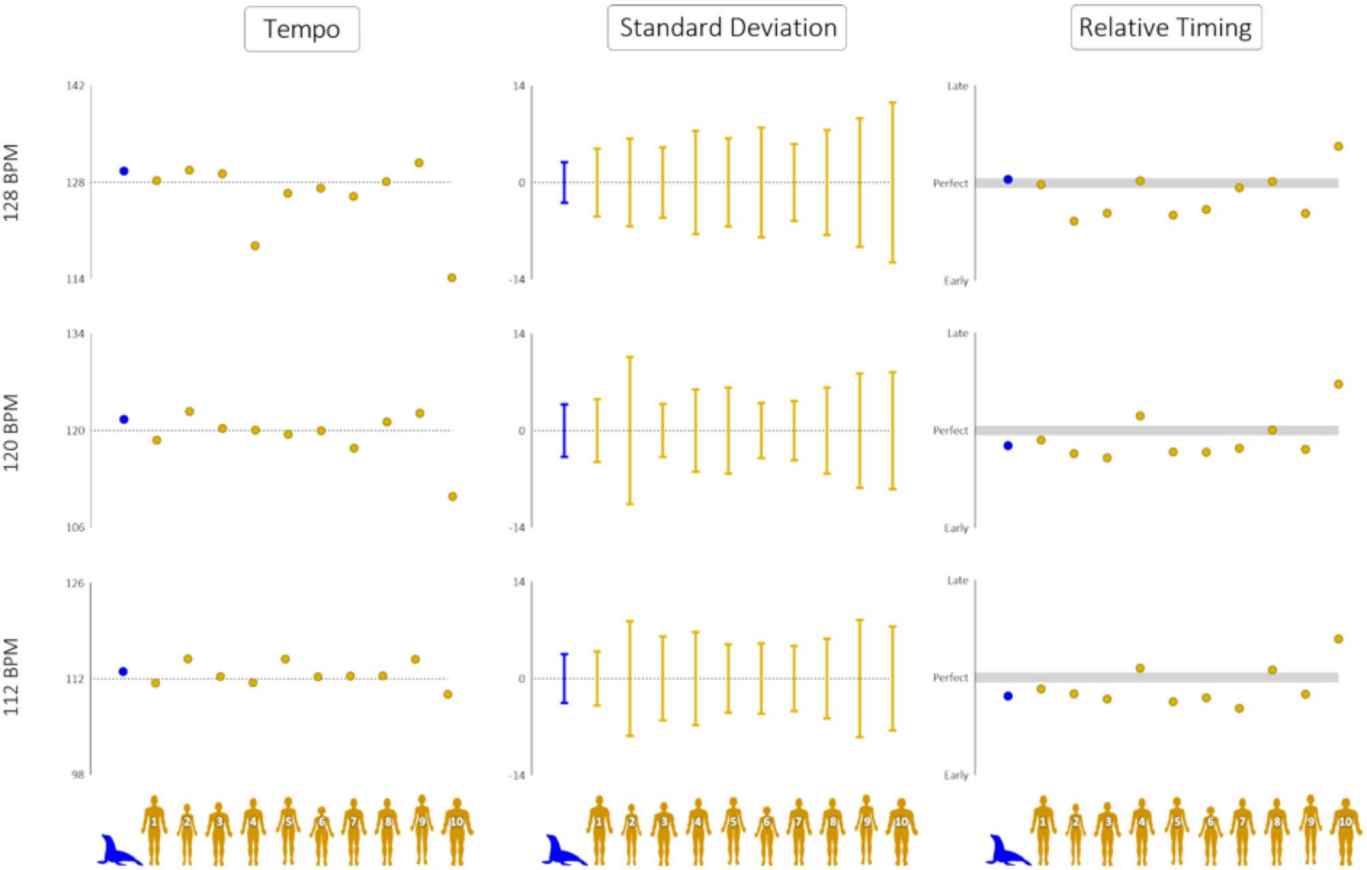
Direct comparisons between sea lion *Ronan* and ten humans across three performance metrics and three tempos. Columns refer to the average of tempos performed, the standard deviation of tempos performed, and movement timing relative to stimulus timing, respectively. Rows refer to the tempos of the stimuli presented. In each instance, the closer the value is to the stimulus tempo, 0, or ‘perfect’, the better the rhythmic performance. *Ronan* matches or exceeds most human subjects in their proximity to perfect performance.

*Ronan’s* entrainment was overall more accurate and less variable than the human subjects tested with the same stimuli (see Fig. 3; Table 2, Supplemental Video S2, https://youtu.be/1zZc18B8ZW0).

**Table 2:**
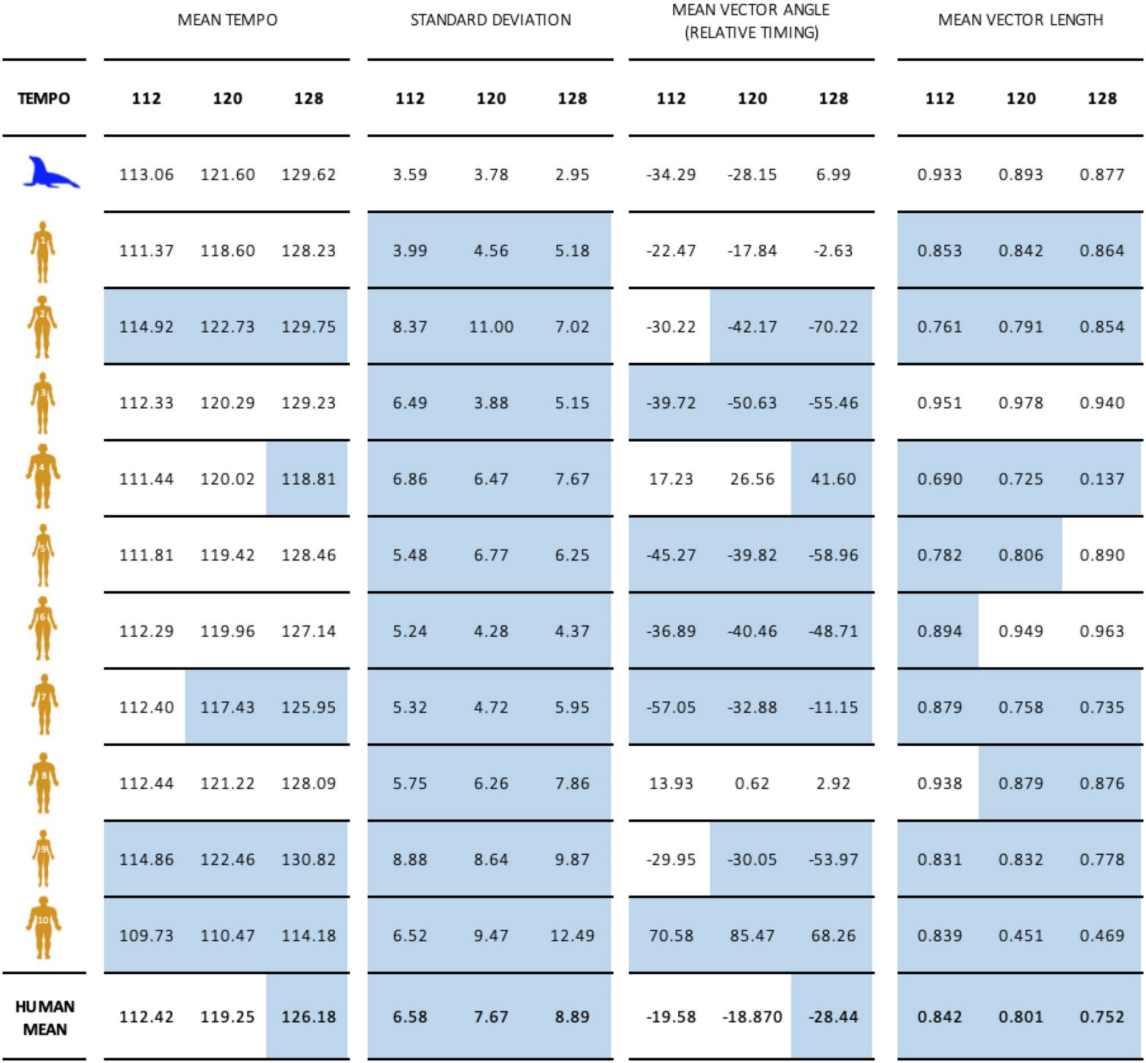
Mean tempo, standard deviation, vector angle (displaying the average timing of the participants’ performed beat-marking movements relative to the beats presented), and vector length (proportion of movements performed in the measured mean vector angle, a mean vector angle of 1.000 indicates all movements fell within reported vector angle) across four exposures (trials) per subject per stimulus tempo. Tempo and vector length are linear averages of the trial means. Standard deviation is the standard deviation of all intervals across trials. Vector angle is the circular average of the trial means. Each instance where sea Lion *Ronan* performed movements that were closer to perfect synchrony than a given human subject is highlighted in Blue. Average human performance data across all conditions is included on the bottom row.

Her mean tempo interval was closer to the actual stimulus rate than 3/10 human subjects at 112 bpm, 4/10 at 120 bpm, and 5/10 at 128 bpm. Ronan’s movement interval standard deviation was lower than all human subjects across all three tempi. Her mean phase angle was closer to 0 (perfect synchronization) than 5/10 humans at 112 bpm, 7/10 humans at 120 bpm, and 8/10 humans at 128 bpm. Finally, her vector length was longer (indicating smaller variation in phases) than 8/10 humans at 112 and 120 bpm and 7/10 humans at 128 bpm. No single human outperformed *Ronan* in all experimental measures: tempo matching, phase matching, standard deviation, and vector length. In a trial-by-trial comparison, sea lion *Ronan’s* mean phase angle was statistically indistinguishable from the human circular mean on 6/12 test trials. Her mean phase angle was closer to zero on 4/6 of the remaining trials (Supplementary Data excel repository: WW Test). *Ronan* and the majority of humans showed a tendency to different phase angle in the second half of individual trials; *Ronan* was more phase negative in the second half of trials, while humans were split between more phase negative and phase positive (Supplementary Data excel repository: Phase Comparison).

At the novel stimulus rate of 112 bpm, *Ronan’s* performed average tempo was 113.1 bpm (± 3.53), her mean phase angle was − 34.3°, and her mean vector length was 0.93. At the familiar tempo of 120 bpm, her performed average tempo was 121.6 bpm (± 3.81), her mean phase angle was − 28.2°, and her mean vector length was 0.89. At the novel stimulus rate of 128 bpm, her performed tempo averaged 129.0 bpm (± 2.94), her mean phase angle was 7.0°, and her mean vector length was 0.88. Across all exposures, *Ronan’s* variability in performed tempo ranged from ± 2.40 to ± 5.05 bpm. *Ronan’s* mean phase angles ranged from − 44.0° to 15.6° across all exposures. *Ronan’s* mean vector lengths ranged from 0.83 to 0.97. *Ronan’s* performance far exceeded chance on 12 of 12 test trials (Rayleigh test, *p* < 0.0001). Further, on all 12 trials, her mean phase was not statistically different from zero (V-test, *p* < 0.0001), indicating auditory-motor synchronization rather than just entrainment.

The human participants performed more variably and less accurately than canonically reported in other rhythmic tasks conducted with small-amplitude movements^30^. Despite the novelty of the task, humans did not show statistical improvement in phase matching over the course of the experiment (Supplementary Data excel repository: Phase Comparison). Across the 10 participants and three tempos, there were only five first-to-last trials that showed significant differences, and three of these were in one participant who showed high variability overall. Importantly, our sample size here was small, and many laboratory studies omit outliers—newer studies suggest more human variability, indicating that the precision of humans has been overstated in the comparative literature (e.g^34^). Mean human tempos for 112, 120, and 128 bpm stimuli were 112.4 bpm (± 6.18), 119.3 bpm (± 6.24), and 126.2 bpm (± 7.34). Across all exposures, the variability in performed tempo in human subjects ranged from ± 2.33 to ± 16.78 bpm. Mean phase angles at 112, 120, and 128 bpm were − 15.9°, -14.7°, and − 21.4°, respectively. Variability in human mean phase angle by trial ranged from − 112.3° to 110.6°. Human mean vector lengths by trial ranged from 0.02 to 0.99. Across all tempi for all human subjects, 113 of 120 trials were significantly different from chance (Rayleigh test, *p* < 0.05), and 101 of 120 trials were not different from a mean angle of 0, indicating perfect synchronization (V-test, *p* < 0.05). Human participants’ mean phase angle at 128 bpm showed greater negative mean asynchrony than at 112 bpm (Watson-Williams test, F = 28.2, *p* < 0.001).

### Model results

We used parameters from human data in this study to generate a Monte Carlo simulation of 10,000 trials of plausible human beat keeping at each of the three tested tempos, and compared *Ronan’s* actual behavior to the simulated human data. Table 3 summarizes the mean output of the model simulating human performance on the performed rhythmic task.

**Table 3:** Mean performance values for the Monte Carlo modeling of simulated human performance at each of the three tempos.

Tempo	Mean interval (s)	Interval Std	Mean phase (°)	Vector length
112	112.19	1.00	-16.13	0.888
120	119.85	1.21	-15.75	0.908
128	128.24	1.12	-27.98	0.917

**Fig. 4:**
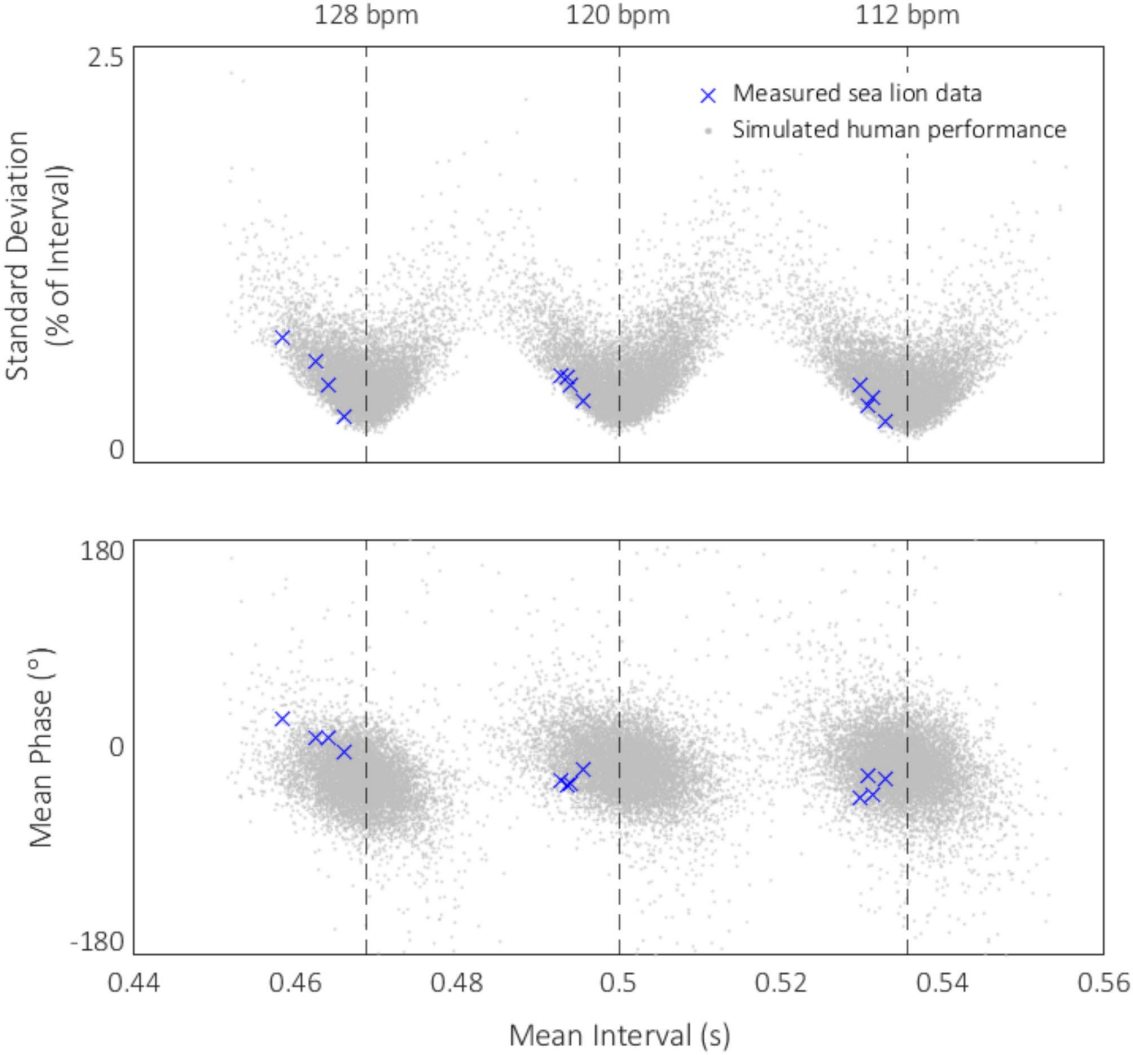
Comparisons between sea lion *Ronan* (blue x, all testing trials) and simulated human (gray dot) performance across three tempi. Simulated trials were created by randomly generating intervals based on the normalized distribution of real human participants’ tempos in trials with vector length > 0.4 and validating their corresponding phases against a similarly generated Von Mises distribution using rejection sampling. 10,000 trials were generated at each tempo, and each trial had a mean interval, mean phase, and vector length (shown here as standard deviation of phase), which are plotted against Ronan’s measured performance. *Ronan’s* data fits within the data cloud, often at the leading edge, suggesting a more consistent performance. In terms of mean phase, Ronan’s mean performance at each tempo was more positive than 7,420 simulated humans at 112 bpm, 6,734 humans at 120 bpm, and 995 humans at 128 bpm.

The model produced U-shaped distributions between mean interval and vector length (see Fig. 4).

In all cases, *Ronan’s* mean tempo was faster than the mean tempo of the model (112 bpm: 113.1 vs. 112.2; 120 bpm: 121.6 vs. 119.9; 128 bpm: 129.0 vs. 128.2), and her phase alignment was closer to 0 (perfect synchronization) than 98% of the simulated human trials.

## Discussion

Following twelve years of intermittent experience with rhythm, sea lion *Ronan* exhibited marked improvement in sensorimotor entrainment consistency, precision, and phase-tempo offsets. For tempos at which *Ronan* was not originally perfect, her phase-angle offsets are now statistically indistinguishable from zero. Between 80 and 128 bpm, *Ronan* now shows no evidence of a phase-tempo relationship. At initial testing, only her performance at 108 bpm, which we’ve theorized might be her native or preferred movement rate, was zero phase. Now there is no statistical difference at any tested rate between *Ronan’s* phase angles and zero phase. Further, *Ronan’s* precision has greatly increased, with standard deviations 30–50% lower than at initial testing. When she was 3 years old, *Ronan* could entrain to a wide range of novel tempos. At age 15 years, she synchronizes with all tempos tested. *Ronan’s* rhythmic apprenticeship has progressed since her initial testing, which no longer represents her full capability.

In the current study, *Ronan’s* performance with increased experience was as good or better than that of ten young adult humans on a closely matched behavioral task, and showed phase angles closer to zero than 98% of 30,000 simulated humans. While some humans outperformed *Ronan* on measures of phase-angle offset, vector length, mean tempo, or standard deviation, no single human outperformed the sea lion on all four. Importantly, *Ronan’s* overall performance was statistically indistinguishable from or better than 10/12 human subjects. Given the current apples-to-apples comparison, there is no justification for suggesting that *Ronan’s* beat keeping capability is inferior to, or categorically different from, that of typical adult humans.

### Phase-tempo relationship

Impressive as *Ronan’s* consistency and precision are in the present study, the reduction of her phase-tempo relationship may be the most notable change from her prior performance. It is possible that this improvement is a byproduct of increased rhythmic maturity—as noted above, humans show a reduction of phase-tempo relationship with increased age and rhythmic experience^26,27^. Training may have also had a specific impact. In the year prior to testing, as *Ronan* engaged again in more consistent rhythm work, her training sessions were shorter, with more variable consecutive bob criteria. This allowed trainers to focus on the precise dynamics of rewarded bobs and bob sequences. In addition, Ronan’s training apparatus was slightly altered. Instead of positioning on a flat surface, *Ronan* performed the task on a slightly inclined ramp. This allowed her head to move up and down freely without touching the ground. Anecdotally, we noticed smoother movement trajectories in this configuration. *Ronan* had previously developed a habit of making a sharp ‘punctuated’ stop at the bottom of her movement trajectory, perhaps to avoid striking the substrate with her whiskers or chin. This may have altered the coupling dynamics of *Ronan’s* movement to the auditory sequences. Although Ronan showed a non-significant tendency toward a more positive mean phase angle for the fastest-paced stimulus in the current experiment, it was notably closer to zero than for similar auditory stimuli during initial testing^11^. There is also evidence in humans that tactile feedback in sensorimotor synchronization can promote stronger phase-tempo relationships^35^. Removing the tactile feedback of touching the experimental apparatus at the low point of each movement cycle may have weakened *Ronan’s* prior phase-tempo relationship.

Importantly, the human subjects in the current study showed a wide range of phase-tempo offsets, greater than those typically reported in button- or key-pressing studies^30^, As discussed above, a greater amplitude movement may create new phase-tempo dynamics or simply amplify those that are already present. We do not believe these large and variable offsets were a product of the novelty of this task to our human participants, as all had experience moving to rhythmic music in these tempo ranges and some pre-exposure to the stimuli presented during testing. Further, there was no evidence of reduction of phase offsets for the human subjects over the course of the experiment, with humans performing no better on the final trial of each tempo than the first. Interestingly, humans did show greater negative mean asynchrony for 128 than 112 bpm. This greater negative phase for faster stimuli might suggest potentially predictive processing by the human participants. We are uncertain why a faster stimulus rate would bring out this shift in strategy. Perhaps a stimulus duty cycle faster than preferred movement rates creates a subjective feeling of being rushed, precipitating an over-correction in phase. Test stimuli in the current study were centered around 120 bpm, *Ronan’s* most commonly exposed tempo, and also a common tempo for human dance music. However, there is some evidence that naturalistic, rhythmic human movements tend to fall in the 100–110 bpm range^36^, just below the tempos used in the current study. More studies on human and animal movement at different rates would be useful^37^. In addition, future work should explore entrainment dynamics with a range of gross motor systems, including the human head and neck. Also of note, while sea lion *Ronan* showed a clear trend toward greater negative phase angle on the latter half of trials, human performance here was mixed, with some humans showing the same trend as *Ronan*, some the opposite, and some showing no clear within-trial trend.

While further comparisons of phase angle tendencies in humans and non-humans under different experimental contexts will no doubt be fruitful, we emphasize that *Ronan’s* training has always focused on beat synchrony. We attempt to reinforce her when her head descends to its low point simultaneously with the beat onset. Unlike humans, *Ronan* does not have cultural and developmental experience with different rhythmic phase relationships. While many human laboratory studies emphasize negative mean asynchrony in human entrainment, we should consider to what extent this is a byproduct of testing context and motor systems. Tight synchrony and positive mean asynchrony are also common in natural human rhythmic behavior, and have recently been studied in human drummers, who can “push” the beat (negative mean asynchrony), be “on top of the beat” (tight zero phase synchrony), or “lay back” on the beat (positive mean asynchrony^38^). Each of these rhythmic styles serves a different musical purpose, and all are within the capability of trained humans. This raises an empirical question: could a sea lion or other non-human animal be trained to be more negative or positive in their synchrony? And how would that affect other aspects of their rhythmic behavior? Future studies with more subjects could isolate these phase characteristics with explicit training.

### The role of experience

It is possible that human performance on this arm movement task would improve given substantially more explicit experience. It would be interesting to assess the responses of human subjects on this task before and after extended practice, and then compare those results to sea lion *Ronan’s*. While the specific stimuli were novel to all participants, *Ronan* had more experience matching high-amplitude movements to metronomic sounds. However, we note that as typical young adults, the humans in this study had far more exposure across the lifespan to human music, and thus a more extensive rhythmic apprenticeship, than has *Ronan*. Much of that exposure history has almost certainly involved some gross movement to music, as is common in human dance, both formal and informal. Human performance on this beat keeping task might also be altered with exposure to complex musical stimuli as opposed to simpler sounds. The subjective human perception of “groove” or “pulse” tends to be stronger for musical than non-musical stimuli, which may affect synchronization behavior^39,40^. *Ronan* has successfully synchronized with music previously^9^, but we do not have evidence that she performs better with musical stimuli than with metronomic sounds. In addition, she has been exposed far more regularly to metronomic than musical stimuli. Nevertheless, most of the careful laboratory experiments of human beat keeping to which *Ronan* has previously been compared have used metronomic stimuli as in the current study^6,8,11,12^.

The human subjects in our study showed fairly high between-subject variability compared to those reported in typical laboratory button-pressing tasks^30^. This is unlikely to be due to different levels of past musical exposure, as none of our participants were trained musicians, and canonical laboratory synchronization studies have also sampled from similar groups of college-age adults. We suspect the higher observed variability in performance was due primarily to the task itself, which involved higher amplitude movements than a button-pressing response. Importantly, many button-pressing tasks do not sample from neurodiverse populations and do remove outliers, so are not fully representative of full human variability.

### Comparable mechanisms

Broadly, the indistinguishability of *Ronan’s* performance from the humans in the current apples-to-apples comparison suggests, contrary to recent theoretical^8^ claims, that her beat keeping ability may indeed rely on proximal mechanisms shared with humans. Importantly, in the current study, both *Ronan* and the humans were tested with simple isochronous stimuli. While extensive research into sensorimotor synchronization with such stimuli has been used to draw inferences about more flexible human beat keeping with complex stimuli, there remain theoretical debates regarding the underlying neural processes^41^. Future work should compare human and non-human rhythmic dynamics with comparable movement across a range of complex stimuli. The current study provides a model for making such comparisons, and directly parallels *Ronan’s* initial rhythmic testing. This returns us to the persistent question of just what the mechanisms underpinning flexible sensorimotor synchronization are. Early comparative beat keeping research with birds^10^ led to the vocal learning hypothesis of entrainment, namely that neural adaptations supporting volitional vocal control and vocal production learning^42^ may have led to beat keeping as an evolutionary exaptation^43^. However, leaving human comparisons aside, newer comparative data have demonstrated some rhythmic performance and beat keeping capability in animals with limited vocal learning capacity, such as primates and rodents that rivals much of what has been shown in psittacine birds^44,45^. We agree with Greenfield and Merker^8^ that the vocal learning hypothesis is a stretch. Perhaps most critically, motor plasticity in response to auditory input in most vocal learning species tends to be slow to emerge and developmentally constrained^46–48^. Regardless, despite their apparent natural interest in rhythmic stimuli, vocal learning species have not distinguished themselves in terms of precision and consistency in rhythm-based tasks compared to sea lion *Ronan*.

As a sea lion, *Ronan* is a member of a clade of mammals that does demonstrate some measure of vocal plasticity^49,50^. Some of the phocid seals and the walruses show reasonably good evidence of at least restricted vocal learning abilities. The otariids, however, separated by about 20 million years of evolution, seem to be less flexible. Assessed in the wild, neither fur seals nor sea lions^51^ show evidence of vocal production learning. There are, as of yet, no laboratory studies indicating enhanced vocal plasticity in the otariids. The common ancestor of seals, sea lions, and walruses was a fissiped carnivore descended from terrestrial carnivores^52^. There is limited vocal plasticity in the carnivore lineage^53^, suggesting whatever vocal learning some seals and walrus species demonstrate may have evolved after the split from otariids, possibly as a consequence of respiratory or feeding adaptations^54^. Current neuroimaging approaches allow for assessment of vocal control adaptations at the neural circuit level and could be applied to pinnipeds as they have to primates^55^. Some of the authors of the current study have already used such data to assess neural pathways in post-mortem sea lion brains^56,57^. Although we do not believe adaptations to these brain pathways in pinnipeds will intersect with the core mechanisms of beat keeping, they will be clarifying and are worth pursuing in their own right.

What other mechanisms present themselves? Some have argued that rhythm is fundamental to inter-cellular communication in the nervous system^58^, and, more specifically, that all sensorimotor coordination is underpinned by rhythmic volleys of neural signaling^59^. Given this perspective, perhaps all animals with complex brains come equipped with the basic toolkit for sensorimotor synchronization. In support of this idea, both human performance and *Ronan’s*^12^ performance on auditory-motor entrainment tasks have been modeled as loosely coupled oscillator output. Still, it is true that spontaneous auditory-motor synchronization behavior to arbitrary stimuli is rarely seen in vertebrates, so there are likely important mediating factors. Some of the current authors have addressed what some of these factors might be^19^, including motivation, degree of volitional control over requisite motor systems, and extent of exposure and learning opportunities. There are also outstanding questions regarding the capability of non-human animals to perceive rhythm, in simple and complex stimuli. Here, laboratory work with model animals such as *Ronan* can be informative.

Further, although sea lion *Ronan* is not a candidate for such studies, there is a productive line of neurophysiological work with primates engaged in rhythm learning tasks^60^. Recent studies have assessed whole brain function in primates engaged in rhythmic behavior and suggested a range of central processing regions that may be in play. Notably, the number of implicated brain regions is large, including visual, parietal, premotor, prefrontal, and medial temporal cortex^61^. Current interpretations have emphasized the potential role of covert simulation of the rhythmic sequence across different neural systems^62^.

It will also be informative to attempt to isolate aspects of human rhythmic behavior that are unique (if any such exist). The comparative data, including those in the current study, indicate some animals can perceive and move to a beat in music, and at least one subject–sea lion *Ronan*–can match or exceed human performance in head-to-head sensorimotor synchronization comparisons. However, humans show a range of rhythmic capabilities that have yet to be assessed in non-humans. Perhaps most notably, humans respond rhythmically to complex, non-isochronous rhythmic patterns^63–65^, and in an anticipatory manner Future work should assess non-human animals’ capability to entrain and synchronize to auditory cues beyond isochrony. Although Ronan’s zero phase offsets do suggest an anticipatory, as opposed to a reactive, capability for entrainment, studies of meter and pattern entrainment would further clarify her capability for internally modeling and anticipating a rhythmic stimulus.

The evolutionary mystery of human rhythmic behavior persists. The present study indicates that contrary to recent theoretical claims, laboratory models with carefully trained non-human vertebrate animals can continue to play a role in elucidating the mechanisms of beat keeping behavior.

## Methods

### Participants

The primary subject was a healthy female 15-year-old California sea lion (*Zalophus californianus)* identified as *Ronan* (NOA006602). After repeated stranding events in the first year of life, *Ronan* was placed at Long Marine Laboratory at the University of California, Santa Cruz and trained for cooperative participation in husbandry and research activities using positive (fish) reinforcement. *Ronan* has taken part in experimental studies in physiology^66^, psychoacoustics^67–71^, and memory^72^. At 3 years of age, she participated in sensorimotor entrainment training and testing^11^. In the following twelve years, she continued intermittent entrainment sessions, including a formal study of her response to rhythmic perturbation^12^. Prior to the current study, *Ronan* had taken part in approximately 700 training and testing sessions (containing 3 to 20 trials per session) with rhythmic stimuli presented at rates of 70–143 beats per minute (bpm). However, the bulk of her exposures were conducted with familiar 80 or 120 bpm simple (metronomic) stimulus tracks. Ronan had been exposed to novel tempos or stimuli during 60 of these 700 sessions, predominantly in testing contexts.

This sea lion was maintained at an optimal weight of approximately 80 kg throughout the study, and her diet was not constrained for experimental purposes. She received 3.5–4.0 kg of freshly thawed herring and capelin fish each day, distributed over 5–8 sessions. Vitamin supplementation was provided, and freshwater was continuously available. Ronan participated in up to one rhythmic entrainment session per day, which lasted 5–10 min. During this session, she received whole capelin fish equally proportioned for each trial. This was equivalent to approximately 30% of her scheduled daily diet.

Animal research was conducted without harm in accordance with the relevant guidelines and regulations of the United States. Authorization for animal research was granted by the U.S. National Marine Fisheries Service under marine mammal research permit 23,554. Animal research activities were conducted with the approval and oversight of the Institutional Animal Care and Use Committee at the University of California Santa Cruz. Daily animal care was provided by marine mammal specialists in the research program and an expert attending veterinarian. All methods are reported in accordance with ARRIVE guidelines as applicable to this study^72^.

Ten human participants were opportunistically selected for a study of rhythmic movement. These were undergraduate students from 18 to 23 years of age (M = 21.70), who self-reported as non-musicians with minimal formal exposure and training in music and dance.

Human research was conducted without harm, in accordance with relevant guidelines and regulations of the United States. Human study protocols were reviewed by the Institutional Review Board at the University of California Santa Cruz. Human participants provided informed consent^73,74^ for participation and recording of their arm movements.

### Task configuration

*Ronan’s* performance was evaluated at Long Marine Laboratory in a quiet outdoor enclosure that included a saltwater pool with adjacent haul-out space. During testing, she positioned at a station that consisted of a 0.3 m tall ramp facing away from her pool and toward a smooth HDPE panel placed 0.8 m from the station in the doorway of her enclosure. This configuration allowed ample space for *Ronan* to move her head and neck. Stimuli were played from a Logitech UE Roll 2 Bluetooth speaker linked to a controlling device with audio playlist. This speaker was mounted to the blind directly in front of her position at the station, and the received level of the stimulus at the position of the sea lion was 80 dB SPL. A trainer seated behind the blind cued *Ronan* to and from her station at the start and end of each session and dispensed fish as instructed by an experimenter, who observed the session from a concealed position perpendicular to *Ronan’s* right side during experimental trials. A Hero 11 GoPro Camera captured continuous high-speed (240 fps) video of each session from a position that was 2.4 m to Ronan’s right side, perpendicular to the axis of her body.

A session would begin when *Ronan* was prompted to the station and the trainer moved behind the blind. A given trial began when *Ronan* assumed a relaxed testing position on the ramp of the apparatus, indicating she was ready for the stimulus to be presented. *Ronan* responded to the acoustic cue immediately, moving her head in a continuous up-down manner. Her correct behavior was marked by a conditioned reinforcer (whistle) delivered by the experimenter following a series of head movements that visually matched the tempo of each beat in the rhythmic stimulus (see below). The conditioned reinforcer was followed by a fish reward. *Ronan* returned to her pool following each trial and returned to the station before the next trial would begin, with inter-trial intervals of about 5 s.

The human participants were tested in a quiet room indoors. Each subject was asked to sit in a chair aligned with a vertical 1.1 m high x 1.4 m wide HDPE panel with their preferred arm hanging over the other side. They assumed a comfortable position with their selected arm out of their view and able to move parallel to the panel. Human participants were instructed to keep their eyes on a large “X” on the forward-facing wall in front of them during each trial. The experimenter exited the room during testing, remaining out of view of the participants. High-speed (240 fps) video was obtained by the same camera placed 1.5 m from the panel, perpendicular to the axis of the subject’s arm motion.

Participants were given brief instructions from the experimenter on how they should perform the behavioral task. While holding their preferred arm over the panel and looking straight ahead, they were told to make repeated downward “chop” like movements with their hand such that the low point of each movement coincided with the onset of each stimulus beat. All human subjects received the same instructions, verbatim, and watched a short video demonstration of the motor behavior with no corresponding audio. Before testing, each human participant demonstrated their understanding of the instructions, without an auditory stimulus, to the experimenter, who provided predetermined feedback if necessary.

### Comparison to previous performance

To compare her performance at age 15 y to age 3 y, *Ronan* was exposed to the same metronomic stimuli with oscillating sinusoidal tones from the original study at four discrete tempos: 80, 96, 108, and 120 bpm^11^. These served as both training and testing stimuli. *Ronan’s* regular exposure to these stimuli took place from July 2023 through March 2024, during which time she received 1684 stimulus exposures over 146 sessions. The presentation of these stimulus tempos was intermixed over the trials within each session, with 5–20 trials conducted per session. The training approach was generally consistent with the operant conditioning methods used in the original study^11^. However, a more variable reinforcement criterion was used during this period, requiring Ronan to produce between 10 and 25 consecutive entrained bobs prior to reinforcement.

Towards the end of this training period (February and March 2024), two typical trials presented at each of the four familiar tempos were randomly selected for analysis. The criteria for their inclusion was that each trial included at least 20 consecutive bobs, and trials at each tempo came from the same training session. This matched the criteria of her assessment at these tempos in the 2013 paper^11^. The two trials at each tempo were evaluated using video analysis as described below and the results were combined for comparison to *Ronan’s* performance with the same stimuli in the initial study:*Ronan’s* first exposures to the 96 and 108 bpm stimuli from initial testing^9^. Because the 80 and 120 bpm stimuli were used during the very early stages of training when Ronan was learning to beat match, we used representative trials at these tempos after *Ronan* had been shown to entrain (her 49th exposure to the 80 bpm stimulus, and her 35th exposure to the 120 bpm stimulus).

### Comparison to human performance

Immediately after the training period, *Ronan* was tested head-to-head against the human participants. Each subject was exposed to five distinct isochronous stimuli made up of 25 equally spaced presentations of a novel sound: a snare drum beat. *Ronan* had not previously been exposed to stimuli with this specific percussive sound.

Prior to testing, each subject received some pre-exposure to the snare stimulus at 96 bpm to ensure that a strong novelty effect^75^ would not disturb performance during testing. *Ronan* was exposed to the 96 bpm snare metronome track in two standard training sessions the day before testing. She received six exposures of 6 to 25 snare beats intermixed with her normal training stimuli. She showed no overt response to the unfamiliar stimuli. On the following day(s) of testing, both *Ronan* and the human subjects received the same two ‘warm-up’ trials. This included one presentation of the 96 bpm snare stimulus superimposed with the same inter-beat oscillating frequency used as a component of the training stimuli, and one presentation of the snare stimulus alone. Each presentation included 25 beats of the snare stimulus. As with the training stimuli, a conditioned reinforcer marked *Ronan’s* final response, which was followed by a fish reward. The human subjects received a tone to signal the end of each trial.

Test trials comprised the same snare stimulus presented at rates of 112, 120, and 128 bpm. The 112 and 128 bpm tempos were novel to *Ronan*, but the 120 bpm rate was one with which *Ronan* had extensive practice, and was also a rate used in comparison of *Ronan’s* current vs. initial performance, as discussed above. 120 bpm is also a typical rate for human biological rhythms and popular music^76^. The sequence of trials was the same for each subject. There were four successive blocks composed of one presentation each of the 112, 120, and 128 bpm snare stimulus tracks in a mixed order. This generated a total of 12 test trials per subject. During testing, each trial was separated by 3–5 s. *Ronan* completed the test trials over two sessions with a break in between. Each human subject was offered a break but declined. When the test session was complete, human participants were thanked and given further details on the nature of the study. *Ronan* received a toy filled with fish and ice.

### Video analysis

Videos of the test trials for all participants were reviewed frame-by-frame with the open-source video software VirtualDub2 (version 1.10.5, http://virtualdub2.com) to identify individual movement patterns and timing. Because *Ronan* matched the low point of each head bob to the beat, and human participants were instructed to match the low point of their hand motion to the beat, the timings of the frames in which each subject’s movements were lowest were used for statistical comparison to actual stimulus beat timings. For *Ronan*, the frame in which the front bottom of her chin reached its lowest point in the camera’s field of view, and thus global screen coordinates, was selected to indicate the timing of each bob. For human participants, the frame at which the arm reached its fullest extension at the terminus of downward hand movement was selected to mark the timing of the chops. In rare instances, human participants would still be in the process of downward arm extension from the elbow when they began pivoting their arm upward from the shoulder. Here, the moment of fullest extension was prioritized over the lowest global arm position. The sea lion did not show this type of movement dynamic.

Because the video was recorded at 240 fps, each frame represented 0.004 s. If more than one consecutive frame showed equally low hand position, the first frame was used to mark the timing of the low point. Human participants sometimes brought their hands down with a small bounce such that there was some number of frames where the hand hovered around the low point, moving slightly up and down. In these cases, the first lowest point frame was still used.

The timing of stimulus beat onset and offset was calculated for each test trial from the same video using sound analysis software Adobe Audition (version 25.0, https://www.adobe.com). The beat timings were then input to VirtualDub2 for comparison to movement low points.

For visualization, continuous movement traces for each trial were automatically pulled from videos using a custom interface written in Python 3.12 with the OpenCV package. These traces were not used for primary entrainment analysis.

### Statistical analysis

Using the frame-based movement timing information and the stimulus beat timing, we calculated the intervals and relative phases between the onset of each snare stimulus and the corresponding low point movement within each trial. Mean and standard deviation for both interval and phase were calculated (phase values ($\:{\theta\:}_{i}$) with circular statistics, with corresponding standard deviations depicted as vector lengths ($\:\bar{R}$), and those values were averaged by subject at each tempo and then across subjects. These descriptive values were compared directly between the sea lion and ten human participants by rank ordering. Better synchronization is indicated by a mean participant tempo closer to the stimulus tempo, low standard deviation of participant movement intervals, participant phase closer to zero, and phase standard deviation (vector length) closer to 1. Vector length, which is analogous to a circular correlation coefficient, is typically used as a representation of statistical effect size.

$$\:{\theta\:}_{i}=360\frac{({t}_{bea{t}_{i}}-{t}_{bo{b}_{i}})}{dt}$$

$$\:\bar{R}=\left|\frac{1}{N}{\sum\:}_{n=1}^{N}{e}^{i{\theta\:}_{n}}\right|$$

Test trials were also analyzed with both the Rayleigh test^77^, which gives the probability that a set of phases is not randomly distributed (i.e. entrained) and the V-test^77^, which gives the probability that a set of phases is not randomly distributed AND not significantly different than a specified mean angle (Rayleigh’s test for uniformity versus specified mean angle, i.e. synchronized).

*Ronan’s* current performance at age 3 was also compared to her performance at age 15 using the same measures and statistics as reported in the original study, with the addition of a Watson-Williams test of homogeneity of means to compare phase angles at each tested tempo.

Several other comparisons were run with the Watson-Williams test of homogeneity of means using raw bob/chop phases. Within each human subject, mean phase of the first trial at each tempo was compared to the mean phase of the last trial at that tempo to look for a possible learning effect. Across subjects, the first 12 chops of human trials were compared to the last 12 chops to evaluate a trend of improvement in phase across a trial. Ronan’s mean phase for each trial was compared to the human performance at that tempo. Ronan’s mean performance at each tempo was compared to the mean human performance at that tempo.

### Rhythm modeling

Distributions of human sensorimotor synchronization behavior have been created previously from laboratory button and key pressing data^30,78,79^. However, we were not aware of any comparable large-scale data sets using gross/high amplitude movement as in the current study. Therefore, to compare *Ronan’s* performance to a potentially more broadly representative model of human performance, we employed a modified Monte Carlo computer simulation of 10,000 human beat keeping trials at each of our three test tempos. We based our simulation parameters on the measured human performance from our ten study participants.

Human trials from the current study were screened to remove any where the mean vector length, a measure of phase variability related to standard deviation, was less than 0.4. Then the interval and phase of all arm movements in the remaining trials were pooled by tempo. Published studies of human beat-keeping often demonstrate trends in phase or interval that can be modeled^58,80^. However, due to the differences in motoric task we sought to avoid any assumption of underlying trends. To minimize any effect of such trends in the human data, the individual arm movements were summarized using a sliding window approach with width = 4 arm chops, such that the first window was the summary of chops 1 through 4, the second window was chops 2 through 5, and so on. Within each window, the distributions of intervals and phases were approximated as a normal and Von Mises distribution, respectively, by calculating the interval mean, and interval standard deviation for the normal and phase mean and phase kappa for the Von Mises. Given the maximum trial length of 25 bobs, this process resulted in *n* = 22 windows.

In addition, beat onset times for each simulated trial were predetermined by setting the first beat onset at t = 0 and calculating subsequent onset times based on stimulus tempo. Onsets were calculated for 29 beats to account for possible skipped beats within the simulation.

Simulated data for each trial was created by the following process: the phase of the first chop was randomly generated from the Von Mises distribution of the first phase window to calculate the first chop onset relative to the first beat. For each subsequent chop_*n*_, the interval_n_ (time between chop_*n-1*_ and chop_*n*_) was randomly generated from the normal distribution of the window for chop_*n*_ to get the onset of chop_*n*_. Then the phase of chop_*n*_ was calculated from the onset time of chop_*n*_ and the onset of the nearest beat. To ensure the resulting distribution of simulated phases was similar to the raw underlying distribution, each calculated phase was validated against that window’s phase distribution with rejection sampling. This was repeated until the simulated trial reached 24 chops, at which point the mean phase, interval, and vector length were calculated.

The simulation generated 10,000 trials for each tempo for a total of 30,000 simulated human trials.

Finally, we compared *Ronan’s* data against the simulated distributions. Simulated data sets, both interval and phase, were large enough that we considered them approximately normally distributed without further testing and performed a z-test on each of the mean interval and phase for each of *Ronan’s* trials at each tempo.

## Electronic supplementary material

Below is the link to the electronic supplementary material.

Supplementary Material 1

## Data Availability

Supplementary Data: Trial by trial data for sea lion and human subjects is available in an excel repository On Figshare: https://figshare.com/s/7cc96198564345ffcd47. Also included in the excel repository are tables comparing Ronan’s mean phase angles at each test tempo to those of human participants (WWTest), Ronan and human participant’s mean phase within trials, comparing first half vs. latter half (Phase Comparison), and human performance on first and last trial at each test tempo (Phase Comparison). Supplementary Video 1: https://youtu.be/oen67G5w5S0. Supplementary Video 2: https://youtu.be/1zZc18B8ZW0. Code for bob tracking and rhythm simulation available at: https://github.com/arouse01/BobbingSimulation; https://github.com/arouse01/MotionTracking.
