# Supplementary material for: Sensorimotor synchronization to rhythm in an experienced sea lion rivals that of humans
Source: Sci Rep. 2025 May 1;15:12125. doi: 10.1038/s41598-025-95279-1 (PMC12045976; doi:10.1038/s41598-025-95279-1)
Supplement: Supplementary file 1 — Supplementary Material 1 [file 41598_2025_95279_MOESM1_ESM.docx]

Supplementary Data: Trial by trial data for sea lion and human subjects is available in an excel repository On Figshare: https://figshare.com/s/7cc96198564345ffcd47

Also included in the excel repository are tables comparing Ronan’s mean phase angles at each test tempo to those of human participants (WWTest), Ronan and human participant’s mean phase within trials, comparing first half vs latter half (Phase Comparison), and human performance on first and last trial at each test tempo (Phase Comparison).

Supplementary Video 1: https://youtu.be/oen67G5w5S0

Supplementary Video 2: https://youtu.be/1zZc18B8ZW0

Code for bob tracking and rhythm simulation available at:

https://github.com/arouse01/BobbingSimulation; https://github.com/arouse01/MotionTracking
